# Genetic Improvement in South African Livestock: Can Genomics Bridge the Gap Between the Developed and Developing Sectors?

**DOI:** 10.3389/fgene.2018.00331

**Published:** 2018-08-23

**Authors:** Esté van Marle-Köster, Carina Visser

**Affiliations:** Department of Animal and Wildlife Sciences, Faculty of Natural and Agricultural Science, University of Pretoria, Pretoria, South Africa

**Keywords:** animal recording, developing countries, indigenous livestock, novel traits, smallholder farmers

## Abstract

South Africa (SA) holds a unique position on the African continent with a rich diversity in terms of available livestock resources, vegetation, climatic regions and cultures. The livestock sector has been characterized by a dual system of a highly developed commercial sector using modern technology vs. a developing sector including emerging and smallholder farmers. Emerging farmers typically aim to join the commercial sector, but lag behind with regard to the use of modern genetic technologies, while smallholder farmers use traditional practices aimed at subsistence. Several factors influence potential application of genomics by the livestock industries, which include available research funding, socio-economic constraints and extension services. State funded Beef and Dairy genomic programs have been established with the aim of building reference populations for genomic selection with most of the potential beneficiaries in the well-developed commercial sector. The structure of the beef, dairy and small stock industries is fragmented and the outcomes of selection strategies are not perceived as an advantage by the processing industry or the consumer. The indigenous and local composites represent approximately 40% of the total beef and sheep populations and present valuable genetic resources. Genomic research has mostly provided insight on genetic biodiversity of these resources, with limited attention to novel phenotypes associated with adaptation or disease tolerance. Genetic improvement of livestock through genomic technology needs to address the role of adapted breeds in challenging environments, increasing reproductive and growth efficiency. National animal recording schemes contributed significantly to progress in the developed sector with regard to genetic evaluations and estimated breeding values (EBV) as a selection tool over the past three decades. The challenge remains on moving the focus to novel traits for increasing efficiency and addressing welfare and environmental issues. Genetic research programs are required that will be directed to bridge the gap between the elite breeders and the developing livestock sector. The aim of this review was to provide a perspective on the dichotomy in the South African livestock sector arguing that a realistic approach to the use of genomics in beef, dairy and small stock is required to ensure sustainable long term genetic progress.

## Introduction

The South African (SA) livestock industry is based on a well-established dairy, beef and small stock industry where selection and breeding practices have been in existence for more than four decades. These livestock species are farmed in all nine provinces of South Africa, characterized by diverse biomes ranging from sub-tropical regions with high rainfall and temperatures to more moderate regions with cold winters and snow as well as semi-desert regions with low rainfall, high temperatures and relatively good quality grazing (Mucina and Rutherford, 2006). Of the total percentage of land available for agricultural production, 68.6% is classified as grazing land (DAFF, 2017a) and used for extensive production of meat producing ruminants. Dairy production is either pasture-based in regions such as Kwa-Zulu Natal (KZN) and the coastal regions of the Eastern Cape (EC) with sufficient rainfall for planted pastures, or produced in Total Mixed Ration (TMR) systems in the remaining parts of SA (Williams et al., 2016).

The South African livestock industry contributed R127 288 million to the Gross Domestic product in 2016–2017 with a positive growth of 11.3% with the largest contribution represented by poultry meat. Animal products contributed 46% of income with regard to all agricultural activities (DAFF, 2017a). It is clear that agricultural sector has an important role considering that sufficient food needs to be produced for approximately 55 million SA population (DAFF, 2016). The current trend is predicting further growth of at least 10 million by 2050 (United Nations., 2012), emphasizing the pressure for increased need for animal derived protein production with higher efficiency.

**Figure 1:**
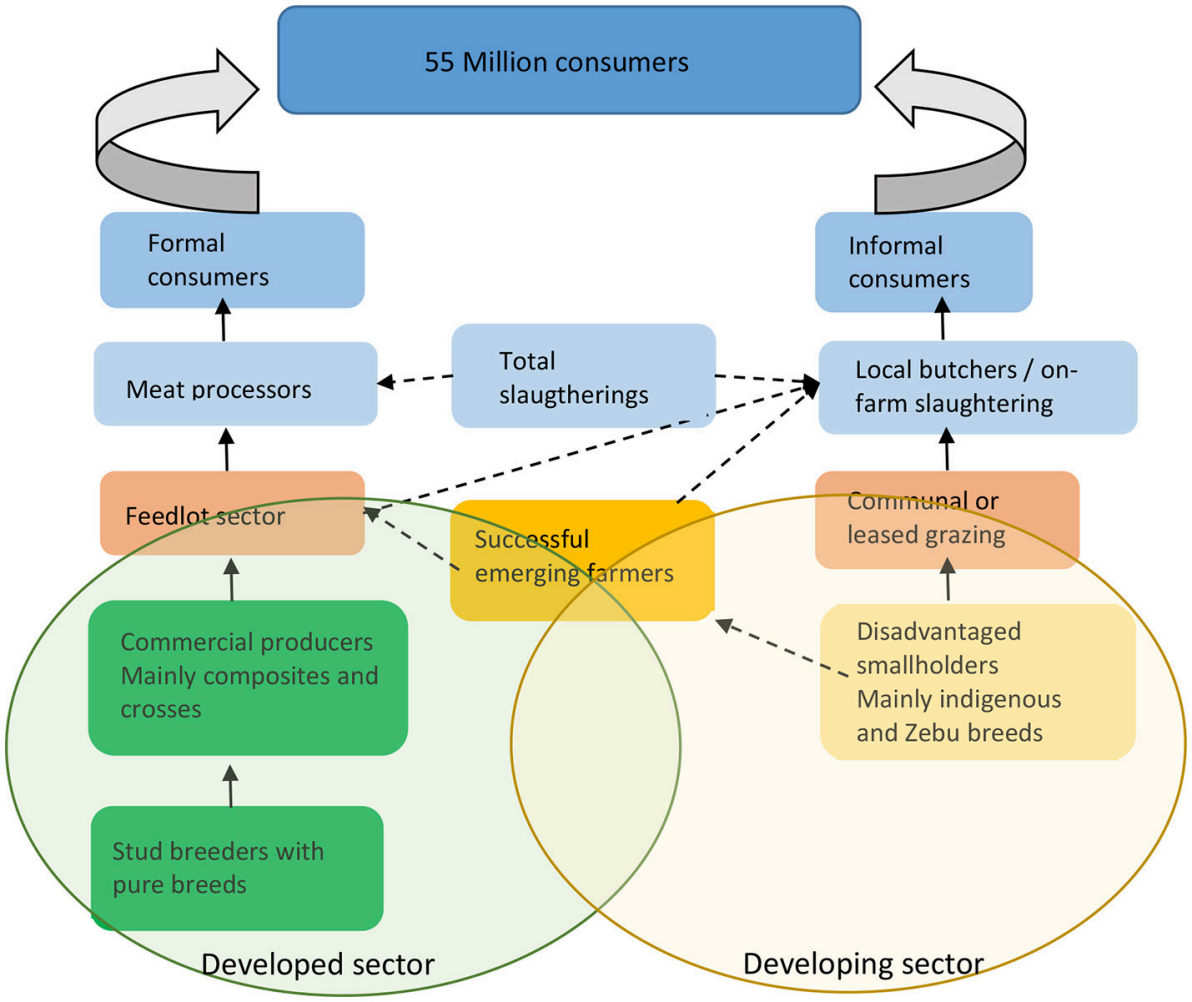
The value chains for the developed vs. developing beef sectors in South Africa (Adapted from http://www.rmrdsa.co.za/REDMEATINDUSTRY/Valuechains.aspx).

The SA livestock industry is characterized by a dual system of a highly developed commercial sector vs. a developing sector. In the developed sector the value chain is differentiated into stud and commercial farmers/producers, feedlots and pigs and poultry companies with good access to abattoirs, product processing and a variety of marketing opportunities. The beef industry value chain is shown an example of this structure in Figure 1. Large and small livestock are primarily individual farms, while poultry and pigs tend to be large companies with vertical integration.

In contrast, the developing sector consists of small holder farmers and livestock keepers within communal systems. There is also a strong presence of a group referred to as “emerging farmers” (more recently referred to as “market-orientated farmers”) in this sector. This group has the potential to become part of the developed commercial sector. The dichotomy of the SA livestock industry is deeply rooted in aspects such as access to land, poor infrastructure, lack of well-structured livestock extensive programs and markets (Mapiye et al., 2018). Development programs, such as the Land Redistribution for Agricultural development (LRAD) and the Independent development Corporation Nguni projects aim to assist the emerging farmer to make the move to commercial farming (Prinsloo, 2008; De Waal, 2014).

In the developed livestock sectors the value chain tend to be fragmented with poor integration of breeding objectives that are set by the stud breeder that markets the genetic material (bulls/rams/buck) versus the commercial cow-calf operation, which in turn produce weaners, and the feedlots who are responsible for finishing and slaughtering. Sheep production follows a similar pattern, but with less feedlot-finishing compared to beef cattle. A similar situation has been described by Pollack (2005) for the beef industry in the United States that results in negative outcomes for selection and long term genetic improvement. The breeding objectives of the stud producer are often not aligned with the needs of the commercial producer, feedlot or end-user (Garrick, 2011). In the developing sector this fragmentation is even more pronounced with a total lack of clear breeding objectives and is further complicated by poor infrastructure and ecological and financial challenges (Mapiye et al., 2018).

Despite a substantial growth in the developing sector over the past two decades with an estimated 1.3 million smallholder farmers, approximately 67% of these farmers are not regarded as emerging commercial operations (DAFF, 2017b; Mapiye et al., 2018). The majority of the smallholder farmers have small herds or flocks where herd sizes could be less than five cows with the majority of these herds being non-descript, crossbred or indigenous cattle, sheep and goats (Mthi et al., 2017; Nyamushamba et al., 2017). Goats for slaughter are mostly marketed directly off the veld through informal trade (Visser, 2018).

Participation in animal recording via national or private services varies significantly among different breeds and between the different livestock species. The majority of beef stud breed societies support animal recording and the use of estimated breeding values (EBVs). In dairy cattle the number of SA stud breeders has declined and commercial producers are moving to automatic recording systems rather than traditional milk recording systems. In the emerging sector the *Kaonafatso ya Dikgomo (KyD)* have been established by the Agricultural Research Council in 2007 to support emerging and smallholder farmers to take part in animal recording. Complete phenotyping however remains a challenge in both the developed sector and even more so in the developing sector with significant adverse implications for genetic evaluations and sustainable genetic improvement.

In 2015 and 2016 state funded genomic programs were established for the SA beef and dairy industries respectively, to set up training populations for moving toward implementation of genomic selection (GS) with the majority of the beneficiaries being stud farmers in the highly developed and technology-driven commercial livestock sector (Van Marle-Köster et al., 2017). The phenotyping of hard to measure traits such as fertility and carcass traits for application in GS and which will realize the most benefit, remains a major challenge (Blasco and Toro, 2014). A further pressing matter is the alignment of breeding objectives within the different sectors to ensure that the traits included in selection programs will benefit all the producers in the value chain. These breeding objectives set within the developed sector should also consider the dissemination of genetic material to the emerging and smallholder farmers in the developing sector. This paper provides a critical review of the dichotomy between the South African livestock developed and developing sectors with regard to the use of genomics in beef, dairy and small stock with reference to the requirements for sustainable long-term genetic progress.

## Historical overview of livestock improvement in South Africa

Since the inception of national animal recording schemes for dairy, beef and small stock in the early nineteen fifties, genetic evaluations for most of these species are routinely performed and stud breeders have access to estimated breeding values (EBVs) as a selection tool. National milk and beef recording date back to 1917 and 1959 respectively, when national recording schemes were managed by the former Animal Improvement Institute (Bergh, 2010). National small stock recording was established in 1956 (Schoeman et al., 2010) with participation by sheep breeders. Angora goat breeders only joined the NSIS in a pilot study in 1983 (Delport and Erasmus, 1984). In Table 1 a summary is provided of the most commonly recorded traits in beef cattle in South Africa.

**Table 1:** Major beef cattle breeds in South Africa and traits recorded (adapted from (Van Marle-Koster et al., 2013).

	**Birth W dir**	**Birth W mat**	**Weaning W**	**Milk**	**12 month W**	**18 month W**	**Post wean W**	**200 d W**	**400 d W**	**Mature W**	**ADG**	**Kleiber Ratio**	**FCR**	**Scrotum circ**.	**AFC**	**ICP**	**Days to calving**	**Calving ease**	**Height**	**Length**	**Carcass W**	**RTU**
**SANGA AND BOS INDICUS**																						
Nguni	x	x	x	x	x	x				x	x	x										
Drakensberger	x	x	x	x			x			x	x	x	x	x	x	x			x	x		
Afrikaner	x	x	x	x	x	x				x	x	x	x	x					x	x		
Tuli	x	x	x	x	x	x				x	x	x										
Brahman	x			x				x	x	x				x							x	x
**EXOTIC BREEDS**																						
Angus SA	x		x	x			x			x	x	x	x	x	x	x			x	x		
Hereford	x	x	x	x	x	x				x	x	x	x	x					x	x		
Sussex	x	x	x	x	x	x				x	x	x	x	x					x	x		
Charolais	x	x	x	x	x	x				x	x	x	x	x	x	x			x	x		
Braunvieh	x	x	x	x	x	x				x	x	x							x	x		
Pinzgauer	x	x	x	x	x	x				x	x	x							x	x		
Limousin	x			x				x	x	x				x							x	x
Simmentaler	x			x				x	x	x				x			x	x			x	x
**COMPOSITE BREEDS**																						
Bonsmara	x	x	x	x			x			x	x	x	x	x	x	x			x	x		
Beefmaster	x	x	x	x	x	x				x	x	x	x	x					x	x		
Santa gertrudis	x	x	x	x	x	x				x	x	x	x	x					x	x		
Brangus	x			x				x	x	x				x			x				x	x
Simbra	x			x				x	x	x				x			x				x	x
Braford	x			x				x	x	x				x			x				x	x

**Figure 2:**
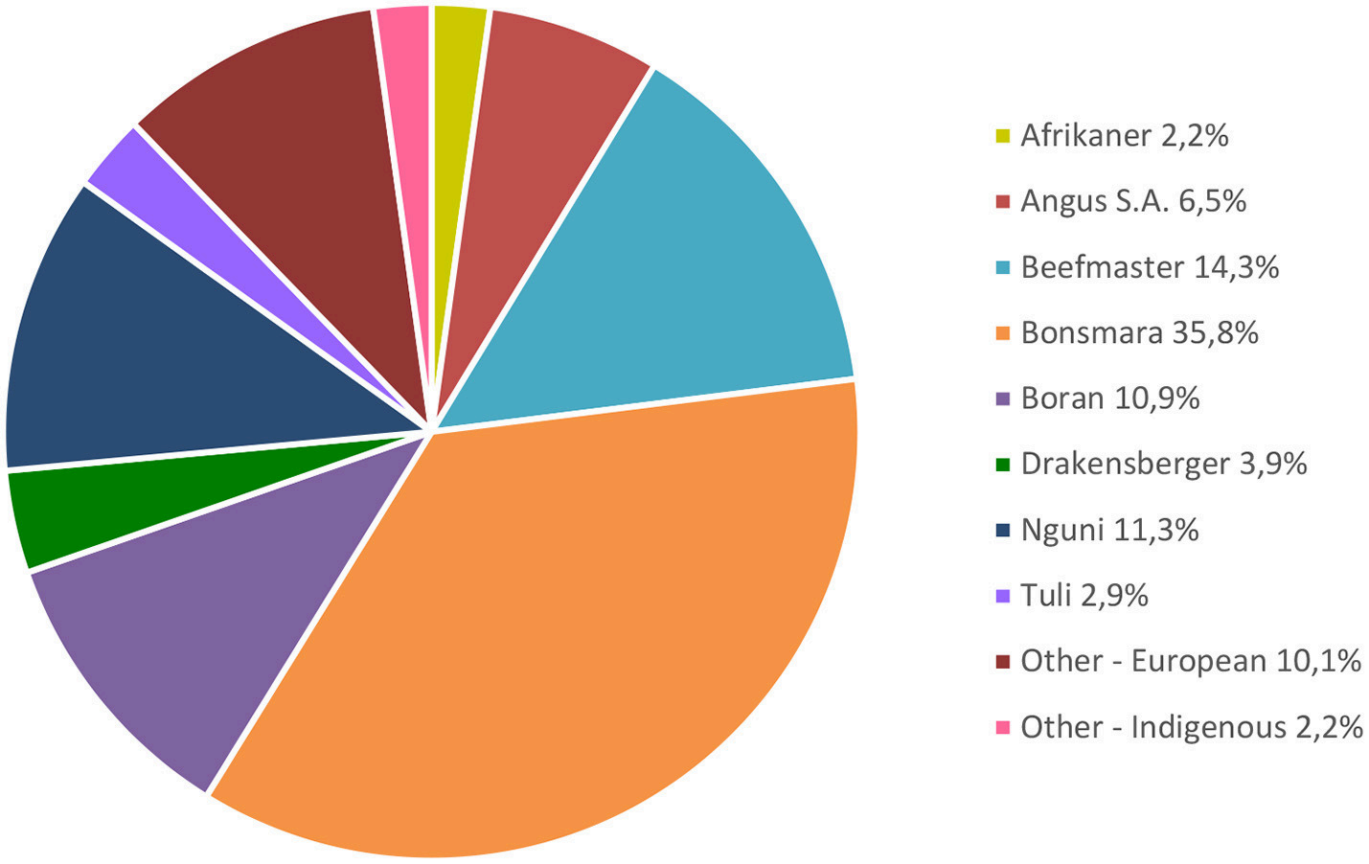
Percentage registered beef cattle participating in Logix Animal recording (SA Stud Book Annual Report, 2016).

South Africa has more than 30 registered beef breeds with large variation among breed societies with regard to participation in recording schemes (Van Marle-Koster et al., 2013; SA Stud Book Annual Report, 2016). Only the locally developed SA Bonsmara composite breed dictates compulsory recording of a number of traits that include fertility, growth and efficiency. In Figure 2 the proportion of registered beef animals in the seed stock industry in South Africa is shown (SA Stud Book Annual Report, 2016). Furthermore, the number of traits recorded varies among the breeds with a larger numbers of phenotypes available for growth traits, compared to limited numbers for fertility or hard to measure traits such as feed efficiency and carcass quality. For most routinely-measured traits of economic importance, there has been a positive trend for adoption of modern selection tools such as EBVs by the livestock producers. Intensive feedlot testing has been popular among some beef breeds with data generated for growth rate, feed efficiency and carcass traits.

Animal recording in the developing sector is limited to the Kaonafatso ya Dikgomo (KyD) scheme where technical advice on health, production and support with recording of animal information is provided. This scheme makes provision for weight recordings at birth, weaning, 12 and 18 months (http://www.arc.agric.za/arc-api/Pages/KyD.aspx).

The dairy industry in South Africa is dominated by the Holstein and Jersey cattle breeds with average herd sizes of approximately 400 cows (Coetzee, 2017). The participation in the national milk recording scheme among commercial producers has been declining over the past decade with only 24% participation (Scholtz and Grobler, 2009) with the trend toward automatic milk systems and recording, especially in larger herds. The dairy industry in SA relies on importation of semen from the best bulls available in the world and the local dairy bull industry has declined significantly. In the developing dairy sector, the majority of farmers own between 5 and 15 cows that produce less than a total of 100 liters milk per day (Muntswu et al., 2017).

**Figure 3:**
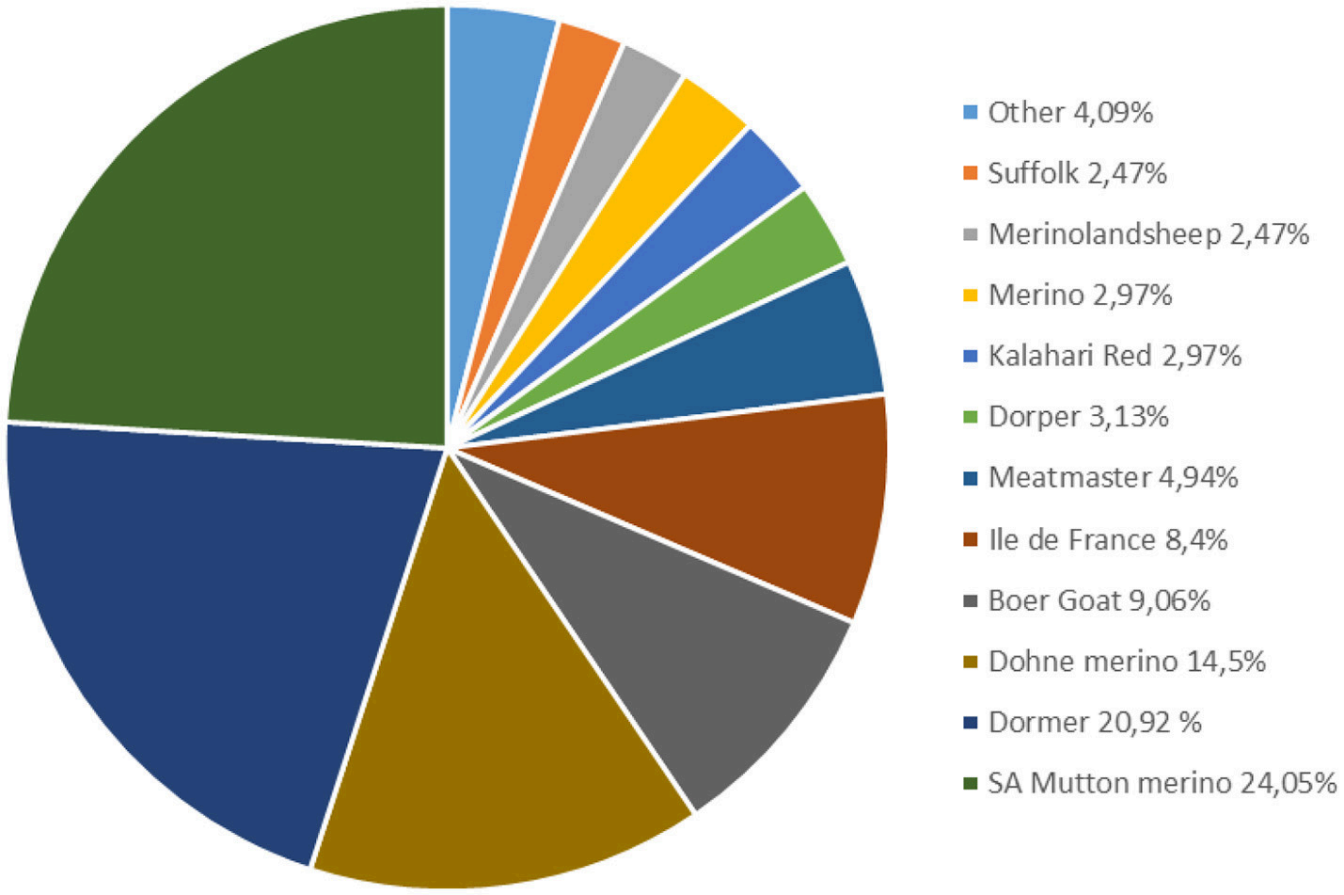
Percentage registered small stock breeds participating in Logix Animal recording (SA Stud Book Annual Report, 2016).

The commercial small stock sector consists of 14 sheep breeds, 3 commercial meat goat breeds and the SA Angora goat breed. The majority of the sheep breeds are farmed under extensive commercial production systems. Participation in animal recording in this sector is limited to a small number of commercial producers (Figure 3), for which genetic analyses are performed.

No recording is performed in the smallholder or communal goat sector (Visser, 2018) which is alarming considering that approximately 60% of goats are kept in these systems and they make a significant contribution to household food security. No genetic improvement in terms of strategic selection or EBV estimation is performed in this sector and genomic applications have been limited to studies on genetic diversity (Mohlatlole et al., 2015; Mdladla et al., 2016).

The challenges for emerging farmers and smallholders are often beyond the scope of the animal scientists and the veterinarian. A number of socio-economic factors such as land issues, financial support and market access are primary constraints in the developing sector (Khapayi and Celliers, 2016). Extension services are also not readily available in all parts of the country to support the number of small holders. Most of these challenges are similar to experiences reported in other developing countries where smallholders (Kosgey et al., 2011) keep beef and dairy cattle. For the emerging and smallholder sectors, genetic tools such as EBVs are unfeasible due to small herds, incomplete recordings for most traits, no parentage recording and insufficient contemporary groups. Different approaches are therefore required to accommodate these farmers to ensure that they will have access to superior genetic material for genetic improvement of their livestock.

## Application of genomics in South Africa

Since the completion of the sheep, beef and goat genomes in 2007, 2009, and 2013 respectively (Fan et al., 2010), followed by SNP marker discoveries (Matukumalli et al., 2009), several applications of genomics have become available for livestock farmers. Over the past two decades, both microsatellite and SNP markers have contributed to the development of diagnostic testing of genetic defects and DNA-based parentage (Van Marle-Koster et al., 2013). SNP arrays are widely applied in routine genotyping for genomic selection in several farm animal species providing an added advantage of using these genotypes for detection and prediction of carriers of genetic defects (Biscarini et al., 2016). Different methods have been reported for prediction that include haplotype-based predictions (Pirola et al., 2013) and discriminant analyses (Biffani et al., 2015). Studies have shown that the accuracy of prediction for the genetic defects could be comparable when using genotypes generated with lower density (Bovine LD) versus a higher density 54K Bovine SNP array (Biscarini et al., 2016). The availability of genotypes furthermore provide the potential for identification of beneficial genes such as the Celtic variant of the POLLED gene for homozygous polled animals (Medugorac et al., 2012).

A number of test facilities are available in South Africa for the diagnostic testing of genetic defects that are relatively cost effective for application in both the commercial and emerging farmer sector (Table 2). DNA technology therefore provides an accessible tool to stud breeders and livestock producers to remove affected animals from their herds. It is also a relatively affordable tool for emerging farmers to solve and manage some basic problems for genetic improvement.

**Table 2:** Diagnostic tests available for ruminants in South African laboratories.

**Diagnostic test**	**Species**	South African Laboratories^*^
DNA profile	Cattle, sheep, goats	Unistel, The Onderstepoort veterinary genetics laboratory, Clinomics, GENEdiagnostics
Parentage	Cattle, sheep, goats	Unistel, The Onderstepoort veterinary genetics laboratory, Clinomics, GENEdiagnostics
3-in-1 DNA/Pompes/CMS	Cattle	Unistel, The Onderstepoort veterinary genetics laboratory, Clinomics
Cytogenetics: 1/29 Translocation	Cattle	Unistel
Double muscling/Myostatin	Cattle	Unistel, Clinomics
Curly calf syndrome	Cattle	Unistel
Polled, scurred, horned	Cattle	Unistel
Bulldog mutation screening	Cattle	Clinomics, Unistel
FreeMartin	Cattle	Unistel

* *Unistel, www.unistelmedical.co.za; Clinomics, www.clinomics.co.za; Veterinary Genetics Lab, www.up.ac.za/the-onderstepoort-veterinary-genetics-laboratory; GENEDiagnostics, www.genediagnostics.co.za*.

For the seed stock industry, accurate pedigree information is essential. Studies performed in South African Angora herds using microsatellite markers (Visser et al., 2011; Garritsen et al., 2015) indicated incorrect and incomplete parentage recording of up to 14%. The largest impact was demonstrated in the accuracy of EBV's with significant re-ranking of the Angora sires (Garritsen et al., 2015). DNA based testing of Boran seed stock in Kenya indicated a 55.2% misidentification of sires and 2.3% for dams (Kios et al., 2012). This situation is not unique to South Africa and Africa as a number of studies reported the adverse effects of incorrect and/or incomplete pedigree information (Visscher et al., 2002; Van Eenennaam et al., 2014).

The use of parentage testing varies among the different livestock species. Approximately 35% of cattle breeders make use of DNA parentage testing on a routine basis, especially larger herds where multi-sire mating is performed. In the small stock industry, group, and over-mating is commonly used resulting in low pedigree accuracies (Visser et al., 2011). Despite the accessibility of DNA parentage testing for sheep and goats, utilization is low due to practical management challenges under extensive production systems. DNA-based parentage verification currently remains limited to the developed livestock sector, mainly due to infrastructural, logistical and financial constraints.

Since the availability of both the ISAG 100 and ISAG 200 panels for bovine parentage validation, more recent studies have highlighted the potential limitations of using a relatively small number of SNP (Strucken et al., 2016; McClure et al., 2018). Due to large-scale genotyping in most world countries the trend is toward large numbers of SNP in combination with different levels of quality control to ensure a high accuracy (McClure et al., 2018). The application of SNP based parentage is only cost-effective if it forms part of routine genotyping. In developing countries such as South Africa where routine genotyping for genomic selection is not standard practice, microsatellite markers are still used for parentage verification. Beef breeds participating in the BGP, will benefit from this added advantage once they engage in routine genotyping.

Genomic technology for application in livestock in South Africa was initiated as recent as 2015 with the founding of the beef genomic program (BGP), followed by the dairy genomic program (DGP) in 2016 (http://www.livestockgenomics.co.za). Both these programs are state funded but have been designed to be driven by the industry with clear objectives toward sustainability with a 10 year period for the beef and 3 years for the dairy industry. The first 3 years for beef cattle have been completed where 16 breed Societies participated and approximately 7,000 samples (hair/semen) have been genotyped with a GGP Bovine150K SNP array. The first genomic enhanced breeding values (GEBV) were published for the SA Bonsmara in August 2017 (Van der Westhuizen et al., 2017) where accuracies were improved between 15 and 30% in traits with low heritability and hard-to-measure phenotypes, such as maternal traits and FCR. Training populations for both dairy and beef cattle in South Africa remain small compared to first world countries, where training populations are replenished by routine genotyping and genomic information used in breeding programs. These programs are however focussed on genomic selection for implementation in the commercial seed stock industry. Several authors reported that the beef industry in general face more challenges with collection of sufficient phenotypes and genotypes compared to dairy cattle (Berry et al., 2016; Piccoli et al., 2017).

Besides commercial application of genomic information in the developed sector of the SA livestock industry, DNA marker technology has been applied for farm animal conservation where the focus has been on indigenous resources. In this regard a number of useful contributions have been made on genetic diversity, inbreeding and population structure of Nguni cattle ecotypes (Makina et al., 2014; Sanarana et al., 2016), Namakwa sheep (Qwabe et al., 2012) and indigenous goats (Mohlatlole et al., 2015; Mdladla et al., 2016). These are all examples of well adapted genetic resources with unique traits that holds potential to be exploited using genomics.

## Novel phenotypes

For many decades the primary focus in commercial livestock production systems was on selection for increased production and traits such as milk yield in dairy cows and weaning and carcass weights in meat producing animals. It is now accepted that the over-emphasis of these traits had adverse effects on health and fertility traits (Miglior et al., 2017) and recommendations to livestock breeders are toward a more balanced approach with breeding goals that include traits associated with fitness, longevity and health.

To make full use of the promise that genomics holds, novel traits have been proposed for most production systems. Dairy cattle pioneered genomic selection (GS) worldwide due to the availability of phenotypic data and DNA available via use of artificial insemination (Wiggans et al., 2011). Due to the intensive nature of dairy production, this was the first industry to recognize the importance of traits associated with sustainability. It resulted in accelerating the process of novel trait identification such as feed efficiency (FE), methane emissions, heat stress and claw health (Miglior et al., 2017; Pryce et al., 2018). Traits such as efficiency, greenhouse gas emissions, and heat tolerance are also of importance in beef cattle and small stock. Examples of novel traits to be considered in selection strategies are presented in Table 3.

**Table 3:** Proposed novel traits for inclusion in selection strategies.

**Trait**	**Heritability**	**References**
**FEED EFFICIENCY**		
RFI	0.00–0.40 0.01–0.40	Egger-Danner et al., 2015 Miglior et al., 2017
CH_4_	0.09–0.35 0.21–0.35	Egger-Danner et al., 2015 Miglior et al., 2017
**CLAW HEALTH**		
Hoof lesions	0.02–0.12 0.01–0.13	Heringstad et al., 2018 Miglior et al., 2017
Lameness	0.02–0.04 0.07–0.15	Egger-Danner et al., 2015 Heringstad et al., 2018
Laminitis	0.06–0.20	Heringstad et al., 2018
**DISEASE RESISTANCE**		
Tick counts	0.03–0.17	Mapholi et al., 2016
Tick resistance	0.15–0.44	Mapholi et al., 2014
Heat stress tolerance	0.17–0.33	Miglior et al., 2017
**UDDER HEALTH**		
Clinical mastitis	0.02–0.09	Egger-Danner et al., 2015
Improved SCC	0.01–0.17	Egger-Danner et al., 2015

Greenhouse gas emissions are closely linked to global warming, and as such has become an important area of research in all ruminant industries. Livestock produce approximately 11–14% of all anthropogenic GHG, with the most significant contribution coming from ruminants (Llonch et al., 2017; Negussie et al., 2017). It is estimated that gastro enteric fermentation by livestock contributes more than 70% of African GHG emissions (Goopy et al., 2018). CH_4_ emissions from developing countries are expected to rise in the next few decades, with Africa predicted to be have the largest CH_4_ emissions (48%) by 2030 (Forabosco et al., 2017). N_2_O emissions are expected to rise concurrently in the same period. Selection strategies to mitigate this problem, includes improvement of fertility, feed efficiency, and animal welfare (Llonch et al., 2017).

Several CH_4_ phenotypes, such as CH_4_ production and CH_4_ intensity have been described (Herd et al., 2013). Individual measurements of these on a large scale are however impractical and expensive. Easy to measure, cost-effective proxies with consistent correlations to CH4 emissions have been identified to mitigate this problem. In a comprehensive review, Negussie et al. (2017) indicated that proxies related to rumen samples (e.g., rumen microbiota, volatile fatty acids) are generally poor indicators of methane emissions. Proxies related to milk yield and components (e.g., fat or protein content) were found to be accurate predictors, with milk mid-infrared (MIR) data showing the most promise.

Using indirect selection, it has been reported that a 24% reduction in CH_4_ emissions can be gained, should fertility rates in dairy cattle be restored to 1995 levels (Llonch et al., 2017). Forabosco et al. (2017) concurred that including traits such as Age at First Calving (AFC), longevity and mortality could also mitigate GHG emissions, as could an increase in litter size. Although directly selecting for more productive animals could decrease GHG levels through a decline in number of animals necessary for the same level of production, it could result in declined animal health and welfare. Care to balance selection pressure must be taken before pursuing such an option. The use of adapted, local genetic resources or crossbred animals could aid in mitigating gas emissions (Forabosco et al., 2017).

In commercial production systems emphases is being placed on improving feed efficiency as it is a notable strategy for reducing GHG emissions (Llonch et al., 2017). Although various measures of feed efficiency are available, e.g., residual feed intake (RFI), residual gain (RG), feed conversion ratio (FCR), and Kleiber ratio (KR), recording is limited to intensive feeding systems (feed lot systems) where individual feed intake can be measured (Berry et al., 2015). Accurate measurements in grazing systems still pose several challenges, especially under extensive production systems. Sensing technologies, such as wireless sensor networks (WSN) (Greenwood et al., 2014) holds great potential for phenotyping grazing animals in their natural environment.

With widespread climate changes facing all aspects of agriculture, breeding of robust animals will become mandatory. High temperatures reduce animal productivity, with a simultaneous rise in parasites and disease pathogens (Taye et al., 2017; Ortiz-Colon et al., 2018). African and locally developed beef cattle have improved thermo-tolerance levels and an increased ability to regulate their body temperature (Taye et al., 2017). High producing dairy cattle are the most susceptible of all ruminant species to high temperatures that result in decreased milk yield (Bernabucci et al., 2014) and feed intake, as well as reduced reproductive efficiency (Garner et al., 2016). Novel traits for measuring heat tolerance are under investigation where Garner et al. (2016) demonstrated the potential for selection of dairy cattle for increased heat tolerance in a simulation experiment. Nguyen et al. (2017) proposed the use of a breeding value for heat tolerance in Australian dairy cattle. The breeding value estimation is dependent on climatic data being known, as well as milk, protein, and fat yields. This is then enhanced with SNP effects, to produce a genomic-only breeding value. It is suggested to use this value in combination with other profit-determining traits. The slick-hair gene has been associated with heat tolerance (Ortiz-Colon et al., 2018) in Slick-haired Holstein calves that had lower vaginal temperatures and respiration rates, mainly due to an increased ability to dissipate heat through sweating. Improved heat tolerance is most likely not due to only the slick-hair gene, but caused by a more complex genetic mechanism.

Lameness is a significant concern in the dairy industry, due to its adverse impact on milk yield, reproductive performance and animal welfare (Randall et al., 2015). Claw health poses challenges with regard to phenotypic recording due to linear indicator traits (locomotion scores). Claw lesions are however not always associated with these type traits (Miglior et al., 2017) and recording through trimming data holds the most potential for direct genetic improvement (Heringstad et al., 2018). Body condition score (BCS) can also be used as an indicator trait of lameness, and has been proposed as a sustainable management intervention (Randall et al., 2015). Maintaining scores of ≥ 2.5 might decrease risk of lameness, especially when used in combination with other risk factors, such as higher parity.

Parasites are a major constraint for livestock production throughout the world, and especially in tropical areas. Alba-Hurtado and Muñoz-Guzmán (2013) reported that losses due to gastrointestinal nematodes (GIN) have been estimated at approximately US$ 400 million per annum in Australia and up to US$ 26 million, US$ 46 million, and US$ 103 million in Kenya, South Africa, and India respectively. The effects of nematode and parasite infection include reduced growth, compromised reproduction, and elevated mortality (Marufu et al., 2011; Guo et al., 2016). Historically, the control of GIN and ticks was largely based on the use of drugs but the development of anthelmintic and acaricide resistance has made this practice unsustainable (Mapholi et al., 2014; McManus et al., 2014). Additionally, the use of drugs is expensive and not affordable by emerging and smallholder farmers (Mpetile et al., 2015). This call for the development of more sustainable, realistic long-term and cost-effective management strategies, such as breeding animals for genetic resistance to parasites (Marufu et al., 2011; Alba-Hurtado and Muñoz-Guzmán, 2013).

Selection for nematode resistance has mainly been based on the use of indicator traits such as fecal egg count (FEC; Riggio et al., 2013), FAMACHA scoring (Van Wyk and Bath, 2002), and body condition score (BCS; Cornelius et al., 2014). The FAMACHA system is based on a standardized chart with illustrations of sheep eyes and membranes in differing hues, indicating varying levels of anemia (Van Wyk and Bath, 2002). While FEC is a difficult to measure trait, especially in rural environments, both FAMACHA and BCS can be used in resource-poor areas as efficient indicators of worm infestation. Easily measured, practical traits for tick resistance include coat characteristics such as hair length and skin thickness (Marufu et al., 2011; Mota et al., 2018). Several studies (Mapholi et al., 2014; Benavides et al., 2015; Mota et al., 2018) have indicated QTL and candidate genes that are associated with resistance to parasites, but it is unlikely that markers will be identified that can serve all breeds. The genetic mechanism for resistance is still not well-understood. Certain indigenous breeds show remarkable resistance to GIN, such as the West African Dwarf goat (Chiejina et al., 2015) and the Nguni to ticks (Marufu et al., 2011). This genetic variation should be exploited in the search for a cost-effective, practical solution to parasite infestation.

Novel traits need to adhere to basic criteria to be useful in breeding strategies. It should be economically important, be heritable with sufficient variation and lastly be practically measurable at a cost-efficient level (Miglior et al., 2017). Some of the traits discussed above, may not yet meet all of the criteria. However, it is crucial to investigate novel traits to make full use of the genetic variation available in the African livestock industry.

## Genomic strategies for sustainable genetic improvement

Genomics has resulted in substantial genetic improvement in most livestock species world-wide. Routine genotyping is performed and genetic evaluations include most traits of economic importance that has been traditionally recorded by breeders. As discussed above, the South African livestock industry is still in infancy with regard to genomic applications and to date limited to the developed livestock sector. In order to design appropriate genomic strategies for the South African livestock industries the dichotomy between the developed vs. developing sector must be addressed as this will influence the long-term application and sustainability of genomics in the SA industry.

The commercial beef and dairy cattle industry have been using available genetic tools such as EBVs, diagnostic tests, and DNA parentage testing in selection programs for genetic improvement (Van Marle-Koster et al., 2013). Genetic improvement has been made in production traits in dairy and beef cattle and sheep breeds using these approaches. To meet the challenges of the Twenty-First century with regard to GHG, feed efficiency, fertility and welfare, novel traits will require emphases in setting breeding objectives and inclusion in current animal recording systems. Application of genomic information holds the most potential in this sector, where state funded programs have been established for genomic selection, providing SA breeders with an additional tool for improving accuracy of selection. Recording of novel traits will incur additional costs for breeders for example using hoof trimmers on a regular basis for claw health in dairy cattle, additional labor for collection of tick counts and using wireless sensor networks (WSN) (Greenwood et al., 2014) and Growsafe/Callen gates technology (Berry et al., 2015) for feed intake and GHG. Although research programs are being established for these novel traits, breeders will have to invest in genomics through extensive phenotypic recordings (Berry et al., 2016) and routine genotyping to reap the benefits.

Routine SNP genotyping of livestock populations in the developing sector will remain a pipe dream for at least a few decades, in the face of more practical challenges such as land availability, droughts, and poverty. In South Africa, both phenotypic and genomic data (in terms of a sufficiently large reference population) pose a challenge for most livestock species kept in smallholder systems. Animal recording is practically non-existent in these extensive systems and measuring of basic traits such as animal weights is problematic with limited equipment and infrastructure. More advanced traits such as direct measuring of GHG emissions pose a greater challenge, due to high measuring costs and expensive infrastructure needed. In addition, most methods to estimate methane production rely on the assumption of *ad libitum* intake, which is often violated in African systems due to tethering and overnight holding of animals (Goopy et al., 2018).

The emerging livestock farmers are in need of good quality male and female genetic stock, which must be supplied by the seed stock breeders. Considering the progress made in the commercial sector over the past three decades, suitable animals (male and female) should be available to already contribute to genetic progress. A study by Mugwabana et al. (2018) have shown that calving rate was positively influenced by using reproductive technologies in emerging and communal farms in South Africa. The adoption of these reproductive technologies (AI) as well as proper animal recording will be cost consideration for these farmers. Farmer co-operatives where bulls and rams are shared, or AI technicians employed can result in genetic improvement in the first generation progeny. In the dairy industry share milking schemes have reported successes where commercial and emerging farmers have formed partnerships (Strydom, 2016). Advantages reported in the study by Strydom (2016) included the access to the livestock skills and technology shared by the commercial farmer, access to markets and gaining business skills. In these systems the basic constraints are overcome, and the emerging farmer can focus on the production, management and selection of the animals. Limited published literature is available of successes of emerging farmers, especially with regard to use of genetic tools and genetic improvement.

Most smallholder farmers make use of indigenous and non-descript crossbreds with no animal recording. The value of adapted indigenous genetic resources in South Africa, which form the basis of smallholder food security, has to a large extent been ignored in the past. Exotic improved breeds often under-perform in the harsh, extensive environments with limited supplementation (Kim et al., 2017). It is ironic that some of the novel traits, such as improved disease resistance and thermo-tolerance that are currently explored in exotic, high-producing world breeds are already present in these local breeds (Kim et al., 2017; Nyamushamba et al., 2017). The greatest benefit of genomics to smallholder farmers might well be the characterisation of their animals, and this benefit may hold great potential in terms of gene introgression into exotic breeds. Using unique haplotypes identified in indigenous breeds, such as hypocretin receptors in trypanotolerance, the *BOLA* complex in tick resistance and heat shock proteins in thermotolerance (Kim et al., 2017) could ultimately benefit commercial producers. Care should however be taken to protect the scarce genetic resource against indiscriminate crossbreeding, which has eroded the unique characteristics of many indigenous breeds.

Genomic technology holds potential for South African livestock breeders. Commercial breeders are becoming aware of the benefits of complete phenotypic recording and routine genotyping. It is important that the research community address the novel traits in the various species to answer the challenges of sustainable livestock production. South African indigenous livestock are valuable resources with unique traits which should be investigated at a genomic level. Genomics will however not bring solutions on the short term to the developing sector and national strategies will be required to first address socio-economic issues including livestock extension support.

## Conclusion

In reviewing the development of the livestock industry in South Africa, it is clear that there is a solid foundation for genetic improvement. Genetic tools and technologies are available but are restricted to application in the commercial sector. In order to reap the full benefits of genomics, commercial breeders will have to invest in recording of novel phenotypes and routine genotyping. The emerging farmers can already benefit from the available superior genetic material, provided that socio-economic factors are addressed by a national strategy. The emerging farming sector is an important link in the dissemination of genetic resources from the commercial farmers to the smallholder farmers. In this way genomics could provide solutions to narrow the current dichotomy in the SA livestock industry.

## Author contributions

All authors listed have made a substantial, direct and intellectual contribution to the work, and approved it for publication.

### Conflict of interest statement

The authors declare that the research was conducted in the absence of any commercial or financial relationships that could be construed as a potential conflict of interest.
